# Identification and characterization of miRNA169 family members in banana (*Musa acuminata* L.) that respond to *fusarium oxysporum f.* sp. *cubense* infection in banana cultivars

**DOI:** 10.7717/peerj.6209

**Published:** 2018-12-21

**Authors:** Shun Song, Yi Xu, Dongmei Huang, Muhammad Aleem Ashraf, Jingyang Li, Wei Hu, Zhiqiang Jin, Changying Zeng, Fenling Tang, Biyu Xu, Huicai Zeng, Yujia Li, Jianghui Xie

**Affiliations:** 1 Key Laboratory of Genetic Improvement of Bananas, Hainan Province, Haikou Experimental Station, Chinese Academy of Tropical Agricultural Sciences, Haikou, China; 2 Key Laboratory of Biology and Genetic Resources of Tropical Crops, Institute of Tropical Bioscience and Biotechnology, Chinese Academy of Tropical Agricultural Sciences, Haikou, China; 3 Department of Plant Breeding and Genetics, University College of Agriculture and Environmental Sciences, The Islamia University of Bahawalpur, Baghdad-Ul-Jadeed Campus, Bahawalpur, Pakistan

**Keywords:** Target gene, miRNA169, Fusarium wilt, qRT-PCR, psRNATarget, Target prediction

## Abstract

MicroRNAs (miRNAs) play an important role in plant resistance to pathogen infections. However, little is known about the role of miRNAs in banana Fusarium wilt, which is the most economically devastating disease in banana production. In the present study, we identified and characterized a total of 18 miR169 family members in banana (*Musa acuminata* L.) based on small RNA sequencing. The banana miR169 family clustered into two groups based on miRNA evolutionary analysis. Multiple sequence alignment indicated a high degree of sequence conservation in miRNA169 family members across 28 plant species. Computational target prediction algorithms were used to identify 25 targets of miR169 family members in banana. These targets were enriched in various metabolic pathways that include the following molecules: glycine, serine, threonine, pentose, glycerolipids, nucleotide sugars, starch, and sucrose. Through miRNA transcriptomic analysis, we found that ma-miR169a and ma-miR169b displayed high expression levels, whereas the other 16 ma-miR169 members exhibited low expression in the HG and Baxi banana cultivars. Further experiments indicate that there were negative relationships between ma-miR169a, ma-miR169b and their targets basing on their expression levels to Foc4 (*Fusarium oxysporum f.* sp. *cubense* tropical race 4) infection in resistant cultivars. But they were low expressed in susceptive cultivars. These results suggested that the expression levels of ma-miR169a and ma-miR169b were consistent with the resistance degree of the banana cultivars to Foc4. The analysis presented here constitutes a starting point to understand ma-miR169-mediated Fusarium wilt resistance at the transcriptional level in banana and predicts possible candidate targets for the genetic improvement of banana resistance to Foc4.

## Introduction

MicroRNAs (miRNAs) are a class of 18- to 22-nucleotide-long noncoding RNAs that play an important role in gene regulation at the posttranscriptional level in plants (Slezak-Prochazka et al., 2010; Bartel, 2004). Extensive research suggests that miRNAs play important roles in various cellular processes, such as organ development, flowering, plant hormone signaling, and plant responses to abiotic and biotic stresses (Samad et al., 2017; Zhang, 2015). Generally, miRNAs silence mRNA molecules by cleaving the mRNA strand into two pieces with completely complementary sequences and destabilizing the mRNA through the shortening of its poly(A) tail with incompletely complementary sequences (Cuperus, Fahlgren & Carrington, 2011). However, *Arabidopsis thaliana* miR172 was determined to be completely complementary to the open reading frame region of the target gene but was found to inhibit protein translation rather than cleaving mRNA (Chen, 2004).

Plant growth and development are influenced by various environmental stresses. In recent years, many studies have demonstrated the involvement of miRNAs in responses to abiotic stresses such as drought, low temperature, high salinity, and nutrient deficiency. Ath-miR394 plays a positive role in the plant response to drought stress by blocking leaf water loss (Song et al., 2013; Ni et al., 2012). The stress-induced expression of miRNAs is used to activate the plant stress signaling system to improve the adaptability of plants to adversity. miR169 is induced by drought stress and functions to increase plant resistance to drought stress in tomato (Zhang et al., 2011), but it decreases drought resistance in Arabidopsis (Li et al., 2008). In addition to controlling targets at the posttranscriptional level in response to abiotic stresses, miRNAs also regulate plant responses to biotic stresses. Tae-miR164 negatively controls the regulation of the target gene *TaNAC21/22,* which is used to improve resistance to stripe rust in wheat species (Feng et al., 2014). NBS-LRR proteins make up the largest class of miRNA-mediated resistance proteins, the genes for which are regulated by Md-miRln11. Md-miRln11 affects the resistance to spot leaf blight in different disease-resistant apple varieties by interacting with its target gene, *NBS-LRR*, and Md-miRLn11 regulates *MdNBS* gene expression to aid in adaptation to pathogen infection (Ma et al., 2014). The osa-miR164-regulated NAC is repressed in response to pathogenic infection (Campo et al., 2013).

The miR169 family is one of the largest conserved miRNA families in the plant kingdom (Sorin et al., 2014). Several studies in recent years have shown that miR169 family members are responsive to abiotic stresses such as salinity, cold, and drought in various plant species (Xu et al., 2016). miR169a is substantially downregulated in both roots and shoots after nitrogen-starvation treatment in Arabidopsis (Zhao et al., 2011). The zma-miR169s and their target *ZmNF-YA* genes exhibit diverse changes in expression patterns after stress treatment in maize leaves (Luan et al., 2015). The overexpression and cleavage of GmNFYA3 mRNA is governed by miR169, which causes reduced leaf area, high water loss, and enhanced drought tolerance in Arabidopsis (Ni et al., 2013). miR169 regulates stress-induced flowering by repressing the *AtNFYA* transcription factor (Xu et al., 2013). miR169/miR169* double mutants are essential for different regulation patterns of *NFYA5* caused by miR169a and miR169l in Arabidopsis (Du et al., 2017). miR169 acts as a negative regulator in rice immunity against the blast fungus *Magnaporthe oryzae* by repressing the expression of nuclear factor NF-YAgenes (Li et al., 2017). However, how miR169 functions in banana to boost immunity remains unclear.

Banana (*Musa acuminata* L.) is the most traded fruit species in the world, and it is widely consumed as a food commodity worldwide (Sreedharan, Shekhawat & Ganapathi, 2013). Banana Fusarium wilt (also known as Panama disease) is caused by *Fusarium oxysporum f.* sp. *cubense* (Foc) and is considered to be the most limiting disease for banana production worldwide. It spreads mainly through the soil and attacks banana plants of all ages, resulting in wilting and yellowing of banana leaves. There are four physiological races of Foc. The history and impact of Fusarium wilt can be summarized with an emphasis on tropical race 4, a “Cavendish”-killing variant of the pathogen that has spread dramatically in banana-planting countries (Ploetz, 2015). Despite substantial documented progress in planting banana-resistant cultivars and implementing biological, chemical, and cultural measures, management is largely restricted to excluding *F. oxysporum f.* sp. *cubense*, and there is no effective prevention method or developed strategy that can prevent Fusarium wilt from spreading (Ploetz, 2015; Guo et al., 2014). Recently, transcriptomic analysis revealed a number of important genes related to salicylic acid signaling transduction (Miao et al., 2017; Li et al., 2013), ethylene (Wang et al., 2015, 2017), and auxin biosynthesis (Song et al., 2016; Hu et al., 2015) involved in the response of banana to Foc infection.

In the present study, we identified and characterized the miR169 (ma-miR169) family members from the banana genome and further investigated their evolutionary relationship, target genes, and expression patterns in various banana cultivars after Foc4 infection. This systematic study will increase our understanding of miRNA169-mediated immunity to *Fusarium oxysporum f.* sp. cubense in banana and lays a foundation for the genetic improvement of banana.

## Methods

### Computational identification of miR169 family members

In our previous study, we identified miR169 family members in banana using small RNA sequencing (Song et al., 2016). The mature miR169 sequences of other plant species were obtained from miRbase database (Release 21.0, http://www.mirbase.org/). The BLAST parameters were the default routine settings of the database. The Latin name of miR169 members of plant species were got from the miRbase though the function of online database “Search by miRNA name or keyword.”

### Prediction of miR169 target genes and functional analysis

The putative target genes for ma-miR169 were predicted using the plant miRNA target prediction online software psRNATarget (http://plantgrn.noble.org/psRNATarget/) with the default parameter settings. We selected the V2 Scoring Schema (2017 release), and applied Musa genomics (Musa acuminate V2, Banana Genome Hub, http://banana-genome-hub.southgreen.fr/home1). psRNATarget is a new web server designed to integrate and analyze reverse complementary matching between target transcripts and small RNAs. Another important function is the evaluation of target-site accessibility through calculation of the unpaired energy utilized to unfold the secondary structure around the miRNA target site in the mRNA. The following parameters were used: penalty for opening gap = 2, penalty for extending gap = 0.5, expectation = 10, penalty for GU pair = 1, penalty for other mismatches = 1, HSP size = 19 and seed region = 2–7 nucleotides. KEGG pathway analysis (Huang et al., 2018) was performed to analyze the metabolic pathways and functions of unigenes (http://www.genome.jp/kegg/).

### Phylogenetic analysis of miR169 family members

Molecular evolutionary analyses were conducted using the MEGA 7.0 program. To evaluate the reliability of the different phylogenetic groups among miR169 family members, a phylogenetic tree was constructed using the maximum likelihood algorithm in the MEGA 7.0 program. Editing was performed in FigTree v1.4.2 (http://tree.bio.ed.ac.uk/software/figtree/). The sequences were aligned using ClustalW. Based on the neighbor-joining method, the molecular evolutionary history was inferred using 1,000 bootstrap replicates to assess the robustness of the tree branches.

### Plant material and growth conditions

Young banana seedlings of four cultivars, named Baxi banana (BX), HongYan banana (HY), FenJiao banana (FJ), and BaoDaojiao banana (BDJ), were obtained. BX is a triploid (AAA) cultivar with high yield, high quality, and the capacity for long-term storage. FJ is also a triploid cultivar, but with a different genotype (AAB), and it is characterized by good flavor, rapid ripening, and tolerance to abiotic stresses. BX and FJ re Fusarium wilt-susceptible cultivars that withers and die after infection. Other cultivars exhibit some resistance to Fusarium wilt, and the infection rate can be controlled at 5–8%. Both are widely cultivated in banana-planting areas. The seedlings used for RT-PCR were tissue culture seedlings that were not exposed to the environment, and the seedling height was approximately six to eight cm. qRT-PCR seedlings needed to be transplanted, and the seedling height was approximately 25–30 cm.

### Pathogen infection and microscopy analysis

All banana species were cultured and propagated separately. Foc4 was confirmed to efficiently infect banana plants and induce disease symptoms. At the seedling stage, the hydroponic suspension was replaced with a Foc4 spore suspension (1.5 × 10^6^ conidia/mL). The pseudostem and roots of the banana were sampled and stored at −70 °C, and part of the postinfection banana was transverse and longitudinally cut for microscopic examination. Macroelements and microelements were supplied throughout the growing stage to maintain growth. The seedlings were grown under a long photoperiod (16:8 h/light:dark).

### Primer design, cDNA synthesis and qRT-PCR analysis

In our previous study, ma-miR169a and ma-miR169b displayed high expression according to the sequencing results. We used the reads per kilobases million values (Additional File 8) to build a heatmap (Becker, Chambers & Wilks, 1988). First, we identified the expression of ma-miR169a, ma-miR169b and their targets. For this purpose, RT-PCR and qRT-PCR primers were designed according to the predicted miRNA and target sequences (Additional File 6). The small RNA samples were converted to miR-cDNA using an RT primer pool with reverse transcriptase. A specific primer pair was designed for each miRNA, after which PCR amplification using a SYBR Premix Ex Taq™ Kit (Takara, Dalian, China) was carried out with a Rotor-Gene 6000 machine (Corbett Robotics, Brisbane, QLD, Australia). Quantification of the relative expression of miRNAs was performed using the ^ΔΔ^CT method, and the U6 gene was used as a control (Song et al., 2018). Quantification of the target was also carried out through qRT-PCR using the actin gene as a control. The primers of the U6 and actin were listed in Additional File 6. The forward and reverse primer sequences flanked the binding region in which the miRNA interacted with its target mRNA.

### Statistical analysis

The quantitative expression data were analyzed statistically using one-way ANOVA followed by post hoc Tukey’s HSD (honest significant difference) tests. The *p-*value < 0.01 was considered statistically significant.

## Results

### Identification and genomic distribution of ma-miR169 family members in banana

**Figure 1:**
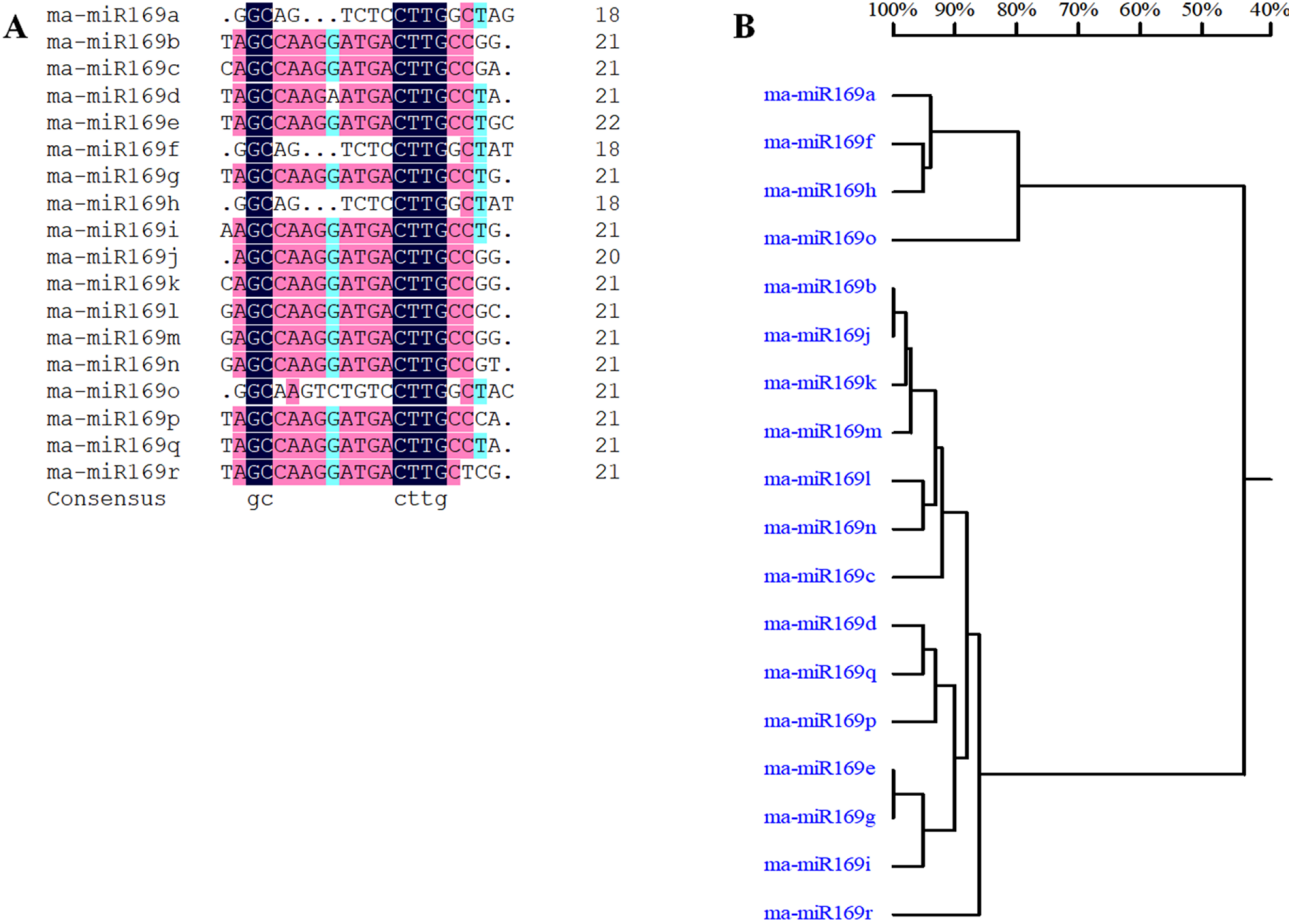
Multiple sequence alignment analysis of ma-miR169 family members. (A) Sequence similarity analysis; (B) evolutionary analysis clustered the ma-miR169 family.

**Figure 2:**
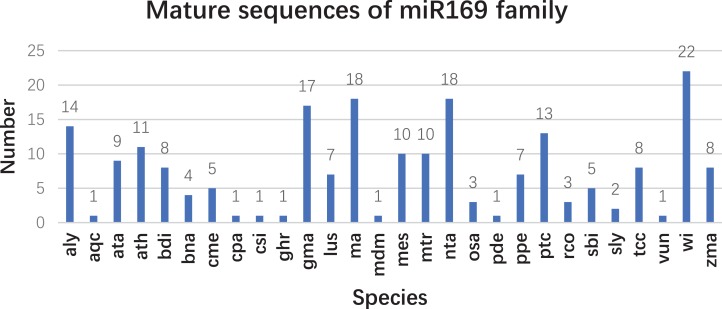
The number of mature sequences from miR169 family members in different species. aly, *Arabidopsis lyrata*; aqc, *Aquilegia caerulea*; ata, *Aegilops tauschii*; ath, *Arabidopsis thaliana*; bdi, *Sorghum bicolor*; bna, *Brassica napus*; cme, *Cucumis melo. cpa (Carica papaya)*, *csi (Citrus sinensis)*, *ghr (Gossypium hirsutum)*, *gma (Glycine max)*, *lus (Linum usitatissimum)*, *ma (Musa acuminata L.)*, *mdm (Malus domestica)*, *mes (Manihot esculenta)*, *mtr (Medicago truncatula)*, *nta (Nicotiana tabacum)*, *osa (Oryza sativa)*, *pde (Pinus densata)*, *ppe (Prunus persica)*, *ptc (Populus trichocarpa)*, *rco (Ricinus communis)*, *sbi (Sorghum bicolor)*, *sly (Solanum lycopersicum)*, *tcc (Theobroma cacao)*, *vun (Vigna unguiculata)*, *vvi (Vitis vinifera)*, and *zma (Zea mays)*.

Based on our previous miRNA transcriptomic data (Song et al., 2016), a total of 18 ma-miR169 members were identified from the banana genome. The sequences of the ma-miR169 family were 18–22 nucleotides in length, and the sequence similarity reached 73.67% (Fig. 1A). Evolutionary analysis clustered the ma-miR169 family members into two groups (Fig. 1B). To identify other various plant ma-miR169 members, a genome-wide search was carried out using the miRNAs database, miRbase (http://www.mirbase.org/cgi-bin/browse.pl). A total of 209 mature miR169 sequences were predicted in 28 plant species (Additional File 1). The numbers of miR169 family members differed greatly among the various species. Compared with the number of miR169 members in other plant species, including 11 in *Arabidopsis thaliana*, three in *Oryza sativa*, eight in *Zea mays*, and 17 in *Glycine max*, the number in banana, 18 was the highest (Fig. 2). The sequences of the miR169 family members were 18–23 nucleotides in length, and the sequence similarity reached 68.85% (Additional File 2), suggesting that the mature sequences of these miR169 members were highly conserved.

### Prediction of ma-miR169 targets

Based on the 18 mature ma-miR169 sequences in banana, we used all the banana gene coding sequences to predict the ma-miR169 targets with the psRNATarget online software (Li et al., 2017). A total of 25 targets were obtained, and the number of targets differed greatly for the different miR169 members. Compared with other miR169 members, ma-miR169h, ma-miR169f, ma-miR169c, ma-miR169d, ma-miR169i, and ma-miR169k had more targets, with 3, 3, 2, 2, and 2 targets, respectively (Additional File 3). In addition, we found a complicated relationship between miRNAs and their targets. ma-miR169b, ma-miR169c, ma-miR169e, ma-miR169g, ma-miR169i, ma-miR169j, ma-miR169k, ma-miR169l, ma-miR169m, ma-miR169n, ma-miR169p, and ma-miR169q had the same target (GSMUA_Achr8T16690_001); ma-miR169c and ma-miR169k had the same target (GSMUA_Achr11T16520_001); and ma-miR169h and ma-miR169f shared three targets (GSMUA_Achr10T02910_001, GSMUA_Achr4T09610_001, and GSMUA_Achr8T20870_001). The targets of ma-miR169a (GSMUA_Achr4T12800_001) and ma-miR169o (GSMUA_Achr2T19800_001) were unique and were not common to other ma-miR169 members; a similar situation was observed for ma-miR169d (GSMUA_Achr8T24960_001 and Achr9T12370_001) and ma-miR169i (GSMUA_Achr1T03730_001 and Achr8T16690_001), which each had two targets.

### Functional annotation of target genes

Within the molecular function category, a large number of target genes were assigned to transcription factors, cellular processes, binding, metabolic processes, and organelles. To understand the biological function of these target genes, we got the metabolic pathway of those targets using the KEGG database (KEGG PATHWAY Database, 2017; http://www.kegg.jp/kegg/). Five target genes (GSMUA_Achr1T03730_001, GSMUA_Achr2T19800_001, GSMUA_Achr4T12800_001, GSMUA_Achr8T20870_001, and GSMUA_Achr11T16520_001) were marked in five metabolic pathways. These pathways included glycine, serine and threonine metabolism; pentose and glucuronate interconversion; glycerolipid metabolism; amino sugar and nucleotide sugar metabolism; and starch and sucrose metabolism (Additional Files 4 and 5).

### Expression profiles of ma-miR169 members in two banana varieties

**Figure 3:**
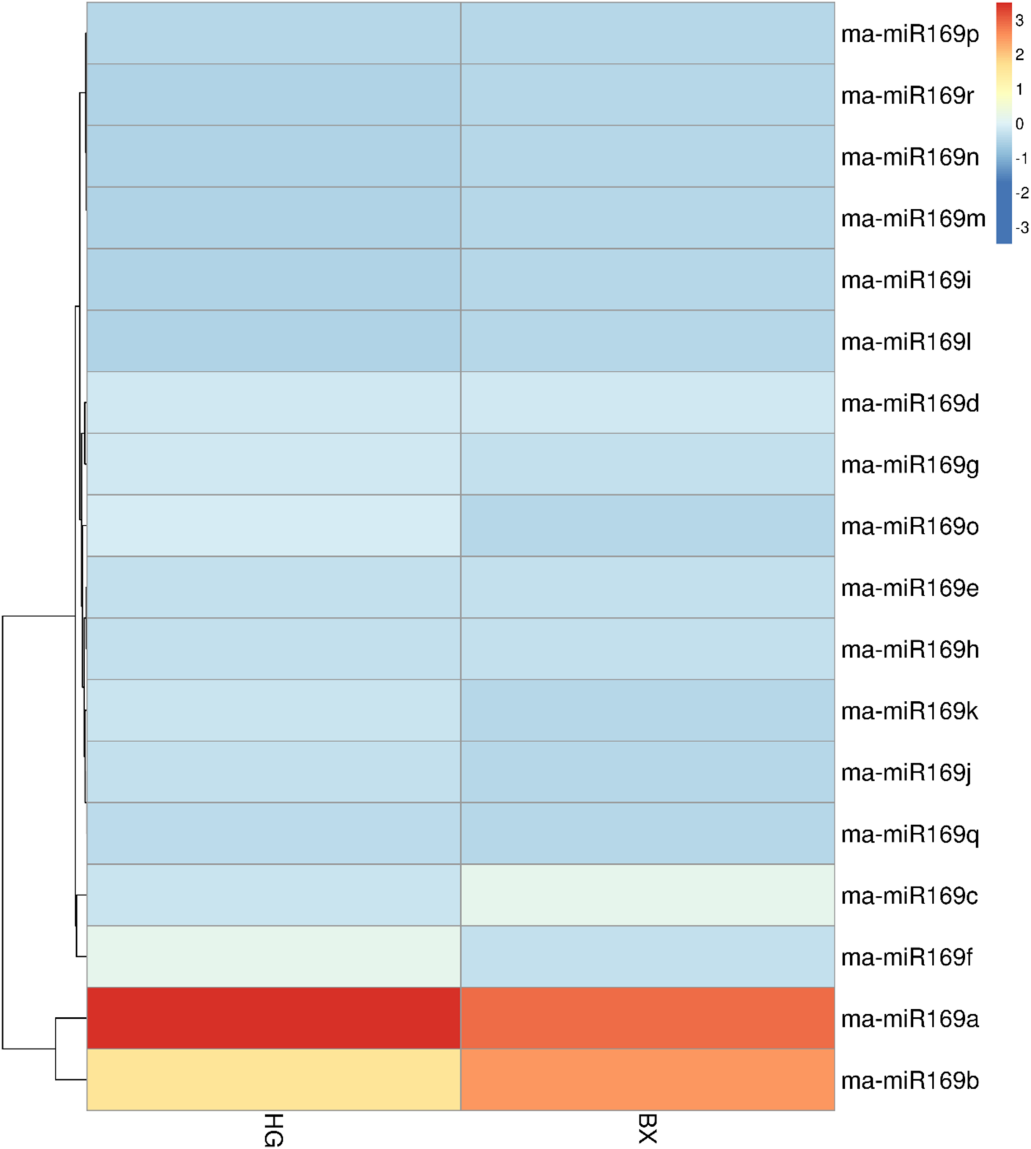
Expression of ma-miR169 family members. The *y*-axis represents ma-miR169 family members; the *x*-axis represents HG (Hainan Gong banana) and BX (Baxi banana).

To investigate the expression patterns of the ma-miR169 family members, roots of HG and BX were collected for transcriptome sequencing (Fig. 3). Generally, when the value of FPKM (Reads per Kilo bases per Million reads) is more than 20, it is considered to be high expression level (Song et al., 2016; Chen et al., 2015), the FPKM of ma-miR169a and ma-miR169b were 68.41 and 36.17, respectively in HG, showing a high expression levels, In contrast, the other 16 ma-miR169 members FPKM values were between 0 and 12.32, exhibited low expression levels. Thus, ma-miR169a and ma-miR169b may be important candidates for the next research.

### The involvement of ma-miR169a and ma-miR169b in the banana response to Foc4

**Figure 4:**
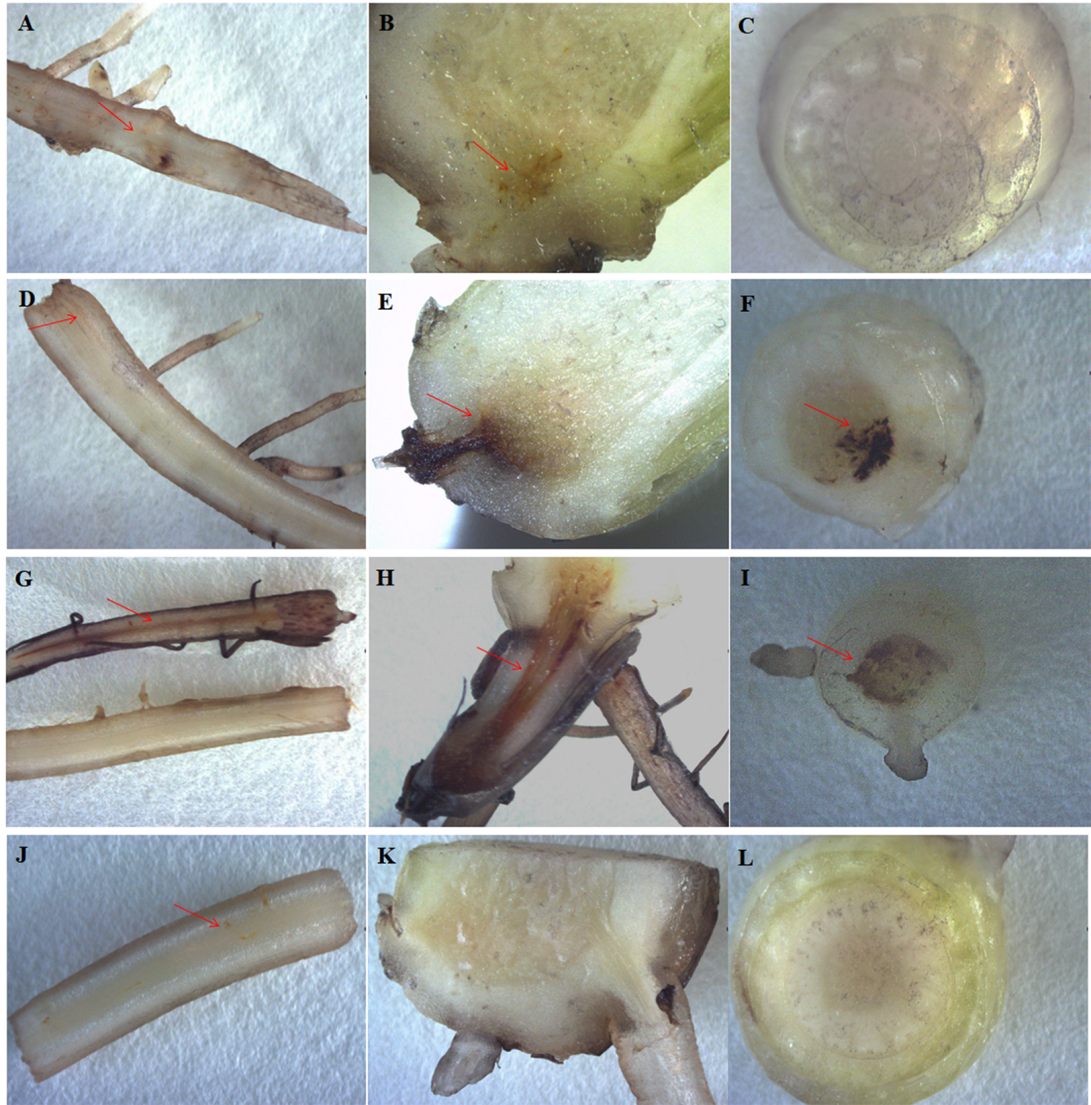
Disease symptoms from four banana cultivars. The roots and pseudostems after treatment with Foc4 are shown. The symbols (A–C) represent HY; (D–F) represent FJ; (G–I) represent BX; (J–L) represent BDJ. The (A), (D), (G), and (J) represent root crosscutting; (B), (E), (H), and (K) represent protocorm longitudinal cutting; (C), (F), (I), and (L) represent pseudostem crosscutting. The red arrow indicates the brown stain. All banana cultivars were examined 30 days after Foc4 inoculation.

The most typical symptoms of the banana infected by Foc4 were brown spots or stains on the roots and protocorms. At 30 days after Foc4 inoculation, no or slight brown staining in the roots and protocorms of HY (Figs. 4A and 4J) and BDJ (Figs. 4B, 4C, 4K and 4L) were observed, whereas there were obvious brown stains in the roots and protocorms of FJ and BX (Figs. 4D and 4G). These results indicate that HY and BDJ are more resistant to Foc4 than FJ and BX.

**Figure 5:**
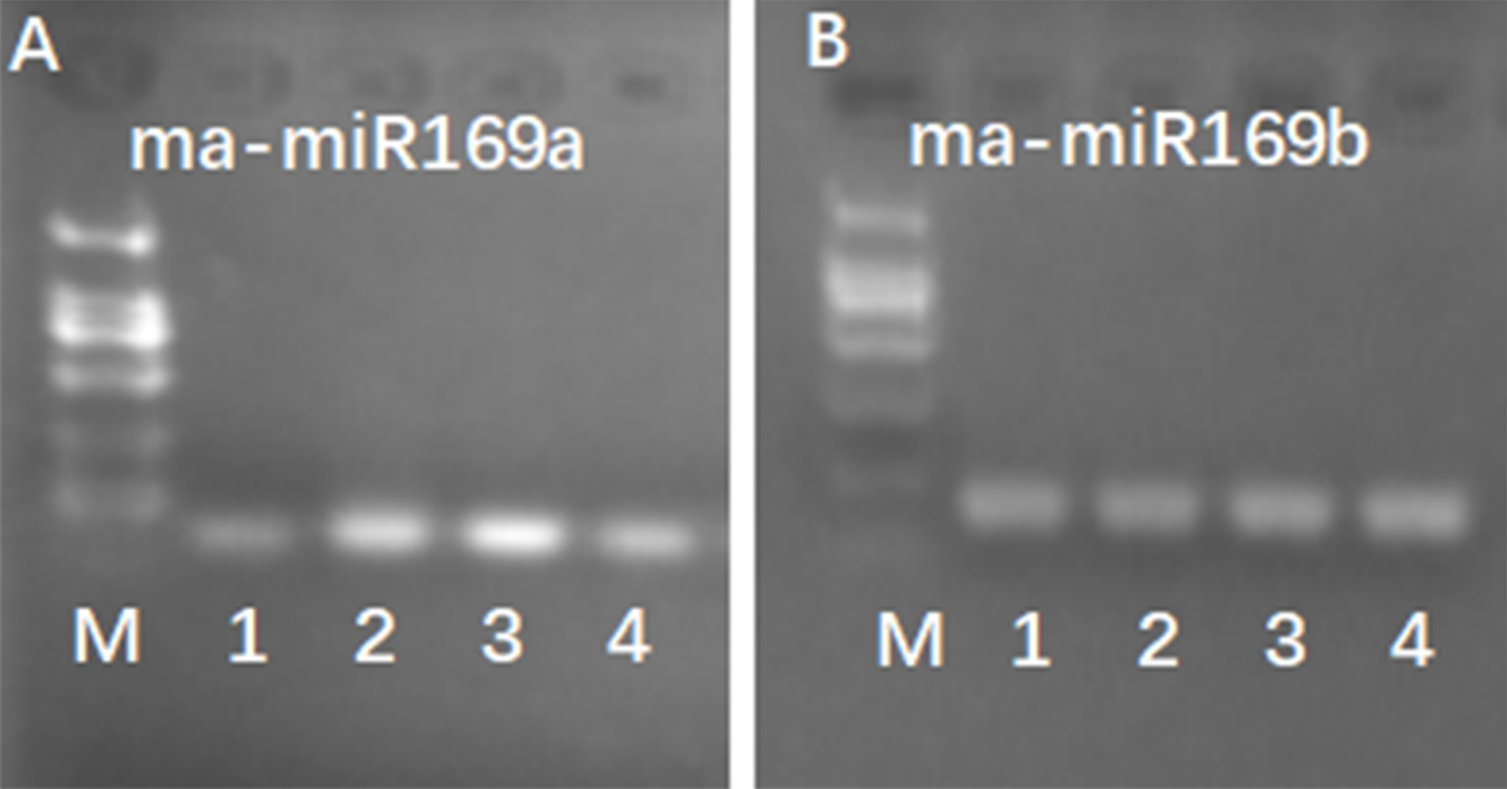
RT-PCR detection of ma-miR169a and ma-miRNA169b expression. (A) Detection of ma-miR169a; (B) Detection of ma-miR169b; M, DL2000; 1, The whole seedling of Hongyan banana; 2, The whole seedling of Fenjiao banana; 3, The whole seedling of Baxi banana; 4, The whole seedling of Baodaojiao banana.

**Figure 6:**
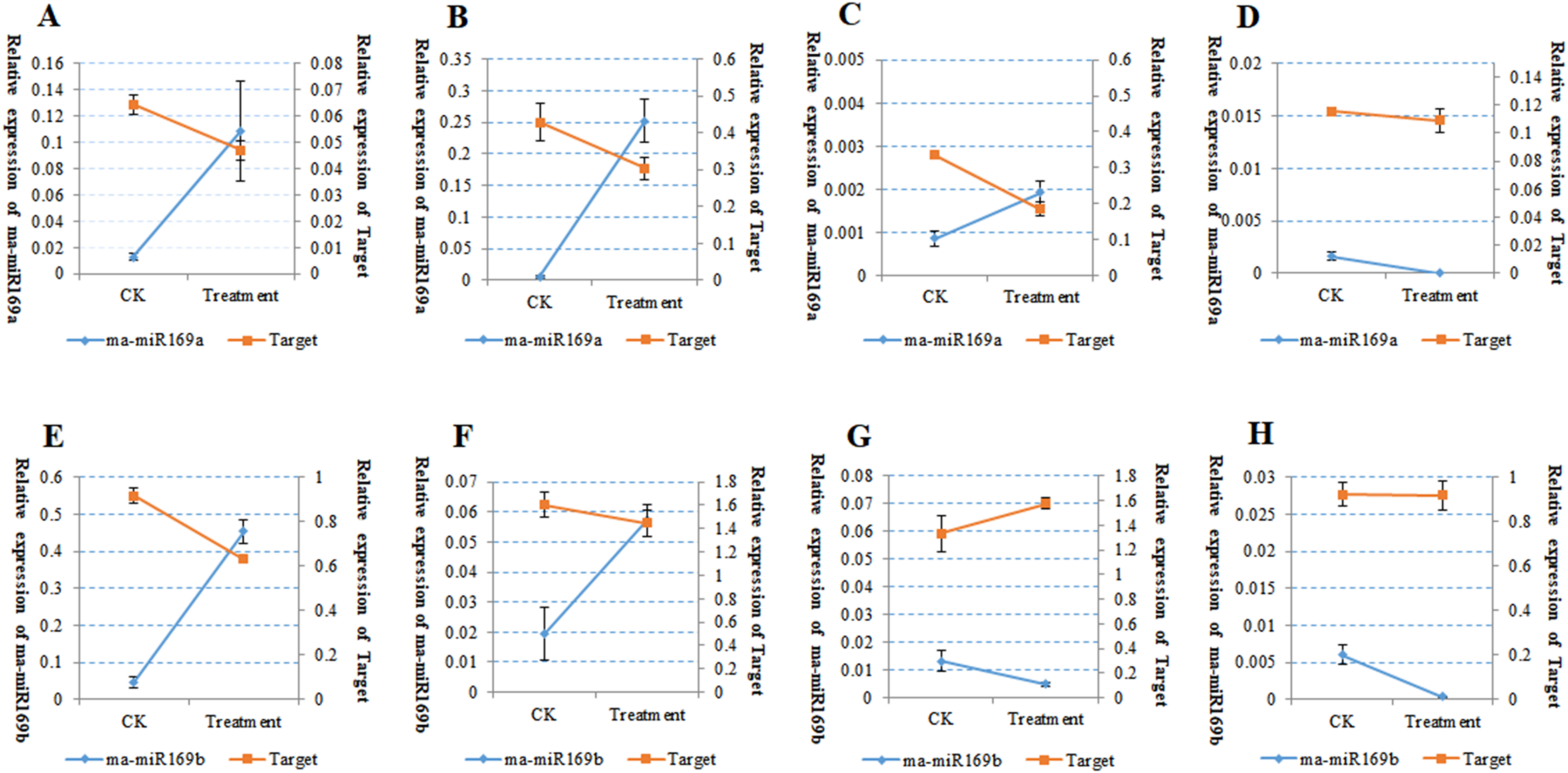
The Expression of ma-miR169a, ma-miR169b and their targets in root in response to Foc4. (A–D) The expression of ma-miR169a and the target gene in different varieties of root; (E–H) The expression of ma-miR169b and the target gene in different varieties of root. (A) and (E) represent HY (Hongyan banana); (B) and (F) represent FJ (Fenjiao banana), (C) and (G) represent BX(Baxi banana), (D) and (H) represent BDJ (Baodao jiao banana). The *y*-main axis represents the percentage of U6 expression, the *y*-subaxis represents the percentage of actin gene expression; the *x*-axis represents control (CK, without Foc4 infection), and treatment (Foc4 infection). Expression was analyzed 30 days after Foc4 infection. The values represent the means, and the error bars represent the standard errors for independent experiments conducted in triplicate.

Because of the obvious phenotypic differences, this set of samples was also used for the expression analysis of ma-miR169a and ma-miR169b. We synthesized reverse transcription primers and qRT-PCR primers (Additional File 7), and cloned those miRNAs into HG and BX, and confirmed the success of the ma-miR169a and ma-miR169b cloning through sequencing (Fig. 5). The expression levels of ma-miR169a and ma-miR169b were significantly increased in the roots of HY and BDJ by Foc4 treatment comparing with controls and their increased fold were approximately 3–43, in contrast, ma-miR169a and ma-miR169b showed low expression or repression in the roots of FJ and BX after Foc4 treatment. Further experiments indicate that there were negative relationships between ma-miR169a, ma-miR169b and their targets basing on their expression levels to Foc4 infection in the roots of HY and BDJ, but they were low expressed in FJ and BX (Fig. 6; Additional File 8). Together, it suggested that the expression characteristics of ma-miR169a and ma-miR169b were consistent with the resistance to Foc4 in banana cultivars.

## Discussion

The miR169 family is one of the largest and most conserved miRNA families in plants (Xu et al., 2016). The miR169 family plays an important role in plant responses to abiotic and biotic stresses (Zhang, 2015). The miR169 members information of plant species can be obtained in the miRbase database, including miRNA precursors, target genes, however, due to the lack of a miR169 reference sequence in the banana genome, the miR169 family of banana is not available in miRbase currently, and little information is known. In this study, 18 miR169 family members were identified in banana, a greater number than that in many plant species (Samad et al., 2017; Zhang & Wang, 2015). These miRNA sequences from the ma-miR169 family are 18–22 nucleotides in length and are comparable to the miR169 family members in other plants (Noman et al., 2017; Li et al., 2016). The nucleotide sequence homology of the ma-miR169 family was observed to be 73.67%, indicating their conserved nature (Ni et al., 2013). Evolutionary analysis divided the banana miR169 family members into two groups, which is consistent with the findings of previous studies classifying miR169 members in poplar (Noman et al., 2017; Liu, Zeng & Tan, 2013). The miRNA target gene prediction algorithm provides a basis for the verification of miRNA function. Several studies have proven that NY-FA family members, as the main targets of miR169 family members, are involved in the development of plant root architecture (Sorin et al., 2014), nodule formation (Zhao et al., 2011), disease resistance (Li et al., 2017), and abiotic stress responses (Ni et al., 2013). Notably, NY-FA family members participate in regulating plant resistance to abiotic and biotic stresses mainly through the ABA pathway (Ding, Zeng & He, 2016; Luan et al., 2015; Zhao et al., 2009).

Overexpression of *HAP2* improves plant resistance to exogenous ABA in aspen and poplar. Overexpression of GmNFYA3 increases plant tolerance to drought and sensitivity to exogenous ABA in soybeans (Ni et al., 2013). The ma-miR169 family has 25 target genes, mainly CK1_casein kinase, single myb histone, plant neutral invertase, 60S ribosomal protein L12, UDP-glucuronic acid decarboxylase, probable mono-galactosyl diacylglycerol synthase, and erythronate-4-phosphate dehydrogenase. We have noted that CK1_casein kinase was predicted to be the target of 12 miR169 family members, suggesting their crucial function. CK1_casein kinases have also been found to be involved in plant responses to stress through the ABA pathway. EL1-like casein kinases function in regulating the stability and function of ABA receptors through phosphorylation (Chen et al., 2018). Casein kinase II mutants (ckb1) exhibit reduced sensitivity to ABA and increased stomatal aperture, leaf water loss, and proline accumulation (Yuan et al., 2017). Plastid casein kinase 2 mutants show reduced ABA sensitivity and thermotolerance (Wang et al., 2014). Together, these findings suggest that CK1_casein kinase may be the target of the ma-miR169 family and may play an important role in the regulation of the ABA pathway. Previous reports have found that cca-miR169a, cca-miR169c, and cca-miR169h are expressed in female flowers of hickory (Sun, Zhang & Wang, 2017). vvi-miR169e, vvi-miR169f, and vvi-miR169g are expressed in the leaf, callus, and stem of grapevine, respectively (Mica et al., 2009). Moreover, miR169 is widely involved in plant resistance to abiotic and biotic stresses. Arabidopsis miR169 can promote leaf dehydration (Ni et al., 2012; Li et al., 2008). miR169-overexpressing tomato plants show improved resistance to drought stress (Zhang et al., 2011). Overexpression of miR169a results in hypersusceptibility to different *Magnaporthe oryzae* strains by inhibiting its targets, NF-YA family members. Overexpression of miR169o leads to susceptibility to bacterial blight in rice (Yu et al., 2018). Tomato miR169 negatively regulates NF-YA5 to enhance tomato resistance to gray mold (Li et al., 2016; Gu et al., 2010).

In this study, we conducted a comprehensive expression analysis of ma-miR169a and ma-miR169b, which exhibited high expression levels in HG and BX banana cultivars. Expression was observed in the roots of four banana cultivars. These cultivars were HY, FJ, BX, and BDJ. However, the function of ma-miR169 in banana responses to Fusarium wilt remains to be explored. In this study, we provided evidence that ma-miR169a and ma-miR169b were induced in the roots of HY and BDJ (two cultivars resistant to Foc4) but repressed in FJ and BX (two cultivars sensitive to Foc4) after Foc4 treatment. There was a negative relationship between ma-miR169a, ma-miR169b and their targets basing on their expression levels to Foc4 infection. Thus, the expression levels of ma-miR169a and ma-miR169b are positively correlated with the resistance of banana cultivars to Foc4, implying a positive role of these miR169 family members in immunity to Foc4 in banana.

## Conclusions

This study identified 18 miR169 family members in banana. Their evolutionary relationships, target genes, and expression patterns in various cultivars after Foc4 infection were systematically analyzed. The ma-miR169a and ma-miR169b can be induced to upregulate by Foc4 and have high expression level in banana resistant varieties. Our study will give a novel sight on miR169’s function which associated with Fusarium wilt.

## Supplemental Information

10.7717/peerj.6209/supp-1 Supplemental Information 1 Supplemental Files. Supplemental file 1: MiR169 family mature sequences of all crops.
Supplemental file 3: The targets of ma-miR169 family.
Supplemental file 4: KEGG Pathway of the targets.
Supplemental file 6: The FPKM values expressed of HG and BX cultivars(Reads per Kilo bases per Million reads values).
Supplemental file 7: The primer pairs for miRNAs and targets comfirmation and quantitative detection.
Supplemental file 8: Expression profiles of mature ma-miR169a, ma-miR169b and their targets in root in response to Fusarium wilt.

10.7717/peerj.6209/supp-2 Supplemental Information 2 Supplemental File 2: The sequences of the miR169 family members. Evolutionary analysis clustered the miR169 family.

10.7717/peerj.6209/supp-3 Supplemental Information 3 Supplemental File 5: The pathways of target genes. 1) Glycine, serine and threonine metabolism, 2) Pentose and glucuronate interconversions, 3) Glycerolipid metabolism, 4) Amino sugar and nucleotide sugar metabolism, 5) Starch and sucrose metabolism.

## Additional Information and Declarations

### Data Availability

The following information was supplied regarding data availability: 1. miRBase: the microRNA database. http://www.mirbase.org/cgi-bin/mirna_summary.pl?fam=MIPF0000012 2. GSMUA_Achr8G16690_001: https://phytozome.jgi.doe.gov/pz/portal.html#!gene?search=1&detail=1&method=4987&searchText=transcriptid:32311987 GSMUA_Achr4G12800_001: https://phytozome.jgi.doe.gov/pz/portal.html#!gene?search=1&detail=1&method=4987&searchText=transcriptid:32297417 3. Designed primers for target genes in the Banana Genome Hub, http://banana-genome-hub.southgreen.fr/primer_designer, after got the RT reverse primer, RT forward primer, and PCR forward primer. After setting relevant parameters, all primers designed were verified experimentally, and then the ones with good specificity are available in Additional File 7.
