# Supplementary material for: Identification and characterization of miRNA169 family members in banana (Musa acuminata L.) that respond to fusarium oxysporum f. sp. cubense infection in banana cultivars
Source: PeerJ. 2018 Dec 21;6:e6209. doi: 10.7717/peerj.6209 (PMC6305118; doi:10.7717/peerj.6209)
Supplement: Supplemental Information 3 — 1) Glycine, serine and threonine metabolism, 2) Pentose and glucuronate interconversions, 3) Glycerolipid metabolism, 4) Amino sugar and nucleotide sugar metabolism, 5) Starch and sucrose metabolism. [file peerj-06-6209-s003.pdf]

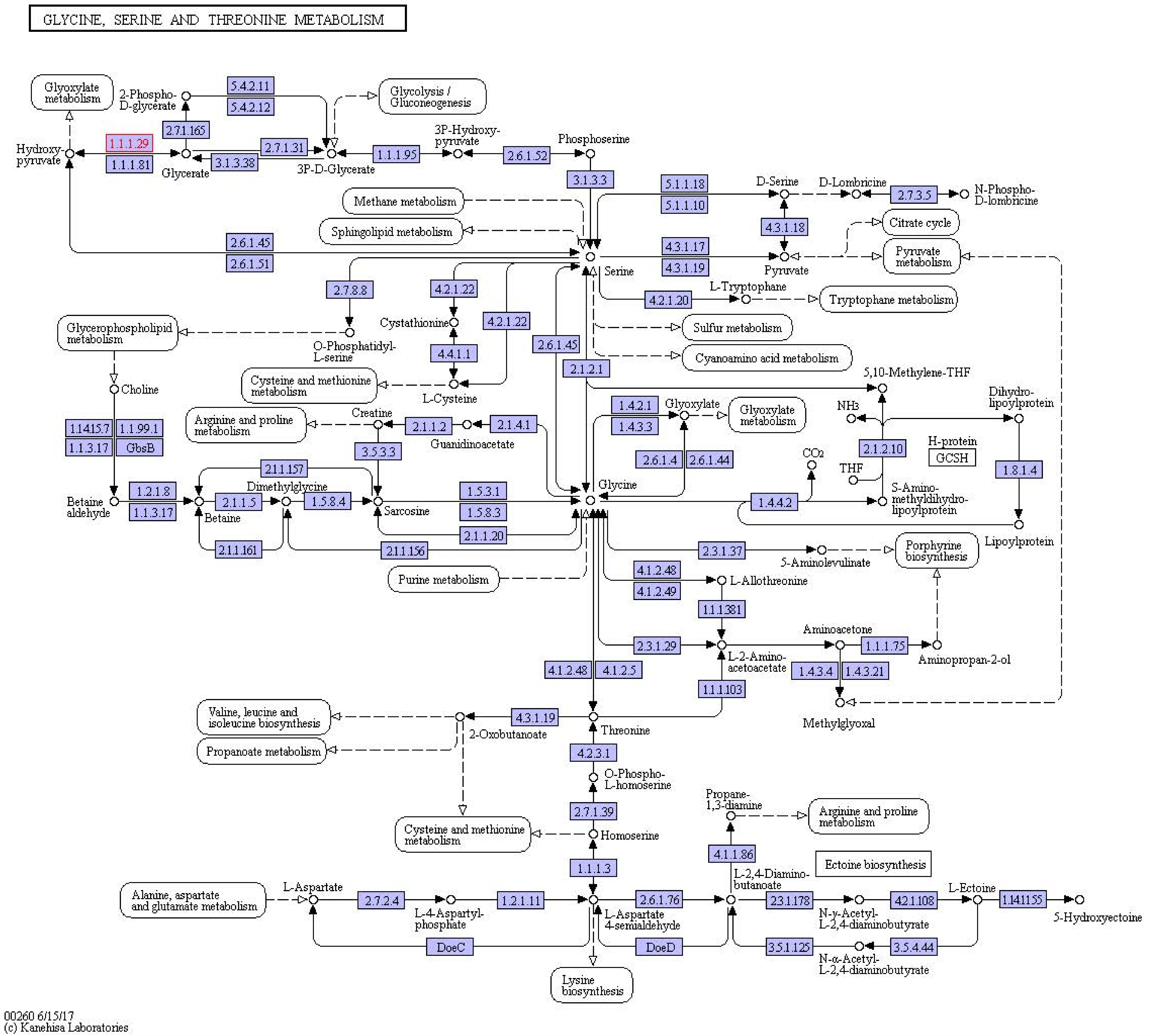
GLYCINE, SERINE AND THREONINE METABOLISM
Glyoxylate metabolism
2-Phospho-D-glycerate
5.4.2.11
5.4.2.12
Glycolysis / Gluconeogenesis
2.7.1.165
Hydroxy-pyruvate
1.1.1.29
1.1.1.81
Glycerate
2.7.1.31
3.1.3.38
3P-D-Glycerate
1.1.1.95
3P-Hydroxy-pyruvate
2.6.1.52
Phosphoserine
3.1.3.3
5.1.1.18
5.1.1.10
D-Serine
D-Lombricine
2.7.3.5
N-Phospho-D-lombricine
Methane metabolism
Sphingolipid metabolism
4.3.1.18
Citrate cycle
2.6.1.45
2.6.1.51
4.3.1.17
4.3.1.19
Pyruvate
Pyruvate metabolism
Serine
2.7.8.8
4.2.1.22
4.2.1.20
L-Tryptophane
Tryptophane metabolism
Glycerophospholipid metabolism
Cystathionine
4.2.1.22
Sulfur metabolism
O-Phosphatidyl-L-serine
4.4.1.1
2.6.1.45
Cyanoamino acid metabolism
Choline
Cysteine and methionine metabolism
L-Cysteine
2.1.2.1
5,10-Methylene-THF
Arginine and proline metabolism
Creatine
2.1.1.2
2.1.4.1
1.4.2.1
Glyoxylate
Glyoxylate metabolism
NH3
Dihydro-lipoylprotein
1.14.15.7
1.1.99.1
Guanidinoacetate
1.4.3.3
1.1.3.17
GbsB
3.5.3.3
2.6.1.4
2.6.1.44
CO2
2.1.2.10
H-protein
GCSH
2.1.1.157
THF
Dimethylglycine
Glycine
1.8.1.4
Betaine aldehyde
1.2.1.8
1.1.3.17
Betaine
2.1.1.5
1.5.8.4
Sarcosine
1.5.3.1
1.5.8.3
1.4.4.2
S-Amino-methyldihydro-lipoylprotein
2.1.1.20
Lipoylprotein
2.1.1.161
2.1.1.156
2.3.1.37
5-Aminolevulinate
Porphyrine biosynthesis
Purine metabolism
4.1.2.48
4.1.2.49
L-Allothreonine
1.1.1.381
Aminoacetone
2.3.1.29
L-2-Amino-acetoacetate
1.1.1.75
Aminopropan-2-ol
4.1.2.48
4.1.2.5
1.4.3.4
1.4.3.21
1.1.1.103
Valine, leucine and isoleucine biosynthesis
4.3.1.19
2-Oxobutanoate
Threonine
Methylglyoxal
Propanoate metabolism
4.2.3.1
O-Phospho-L-homoserine
2.7.1.39
Propane-1,3-diamine
Arginine and proline metabolism
Cysteine and methionine metabolism
Homoserine
4.1.1.86
Ectoine biosynthesis
1.1.1.3
L-2,4-Diamino-butanoate
Alanine, aspartate and glutamate metabolism
L-Aspartate
2.7.2.4
L-4-Aspartyl-phosphate
1.2.1.11
L-Aspartate 4-semialdehyde
2.6.1.76
2.3.1.178
N-γ-Acetyl-L-2,4-diaminobutyrate
4.2.1.108
L-Ectoine
1.14.11.55
5-Hydroxyectoine
DoeC
DoeD
3.5.1.125
N-α-Acetyl-L-2,4-diaminobutyrate
3.5.4.44
Lysine biosynthesis
00260 6/15/17
(c) Kanehisa Laboratories

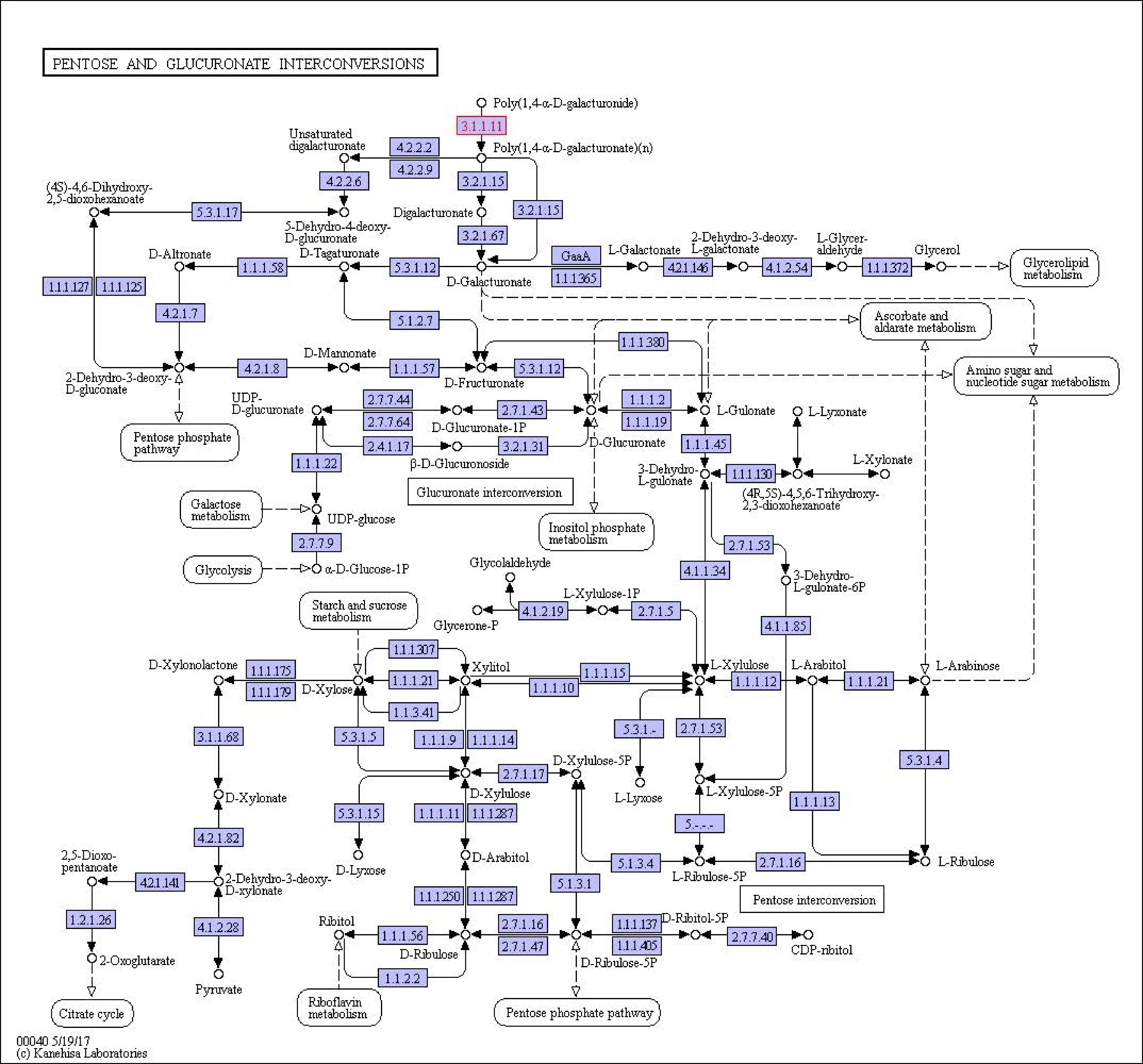

PENTOSE AND GLUCURONATE INTERCONVERSIONS
Poly(1,4-α-D-galacturonide)
3.1.1.11
Poly(1,4-α-D-galacturonate)(n)
Unsaturated digalacturonate
4.2.2.2
4.2.2.9
4.2.2.6
3.2.1.15
3.2.1.15
(4S)-4,6-Dihydroxy-2,5-dioxohexanoate
5.3.1.17
5-Dehydro-4-deoxy-D-glucuronate
Digalacturonate
3.2.1.67
D-Altronate
D-Tagaturonate
1.1.1.58
5.3.1.12
D-Galacturonate
GaaA
1.1.1.365
L-Galactonate
4.2.1.146
2-Dehydro-3-deoxy-L-galactonate
4.1.2.54
L-Glycer-aldehyde
1.1.1.372
Glycerol
Glycerolipid metabolism
1.1.1.127
1.1.1.125
4.2.1.7
5.1.2.7
Ascorbate and aldarate metabolism
1.1.1.380
Amino sugar and nucleotide sugar metabolism
2-Dehydro-3-deoxy-D-gluconate
4.2.1.8
D-Mannonate
1.1.1.57
D-Fructuronate
5.3.1.12
UDP-D-glucuronate
2.7.7.44
2.7.7.64
2.7.1.43
D-Glucuronate-1P
1.1.1.2
1.1.1.19
L-Gulonate
L-Lyxonate
Pentose phosphate pathway
2.4.1.17
β-D-Glucuronoside
3.2.1.31
D-Glucuronate
1.1.1.45
1.1.1.22
3-Dehydro-L-gulonate
1.1.1.130
L-Xylonate
(4R,5S)-4,5,6-Trihydroxy-2,3-dioxohexanoate
Glucuronate interconversion
Galactose metabolism
UDP-glucose
Inositol phosphate metabolism
2.7.7.9
2.7.1.53
Glycolysis
α-D-Glucose-1P
Glycolaldehyde
4.1.1.34
3-Dehydro-L-gulonate-6P
L-Xylulose-1P
4.1.2.19
2.7.1.5
Glycerone-P
Starch and sucrose metabolism
4.1.1.85
1.1.1.307
D-Xylonolactone
1.1.1.175
1.1.1.179
Xylitol
1.1.1.15
L-Xylulose
L-Arabitol
L-Arabinose
D-Xylose
1.1.1.21
1.1.1.10
1.1.1.12
1.1.1.21
1.1.3.41
3.1.1.68
5.3.1.5
1.1.1.9
1.1.1.14
5.3.1.-
2.7.1.53
5.3.1.4
D-Xylulose-5P
2.7.1.17
D-Xylulose
L-Lyxose
L-Xylulose-5P
D-Xylonate
1.1.1.13
5.3.1.15
1.1.1.11
1.1.1.287
5.-.-.-
4.2.1.82
D-Arabitol
2,5-Dioxo-pentanoate
D-Lyxose
5.1.3.4
2.7.1.16
L-Ribulose
4.2.1.141
2-Dehydro-3-deoxy-D-xylonate
L-Ribulose-5P
5.1.3.1
1.1.1.250
1.1.1.287
Pentose interconversion
1.2.1.26
4.1.2.28
Ribitol
2.7.1.16
1.1.1.137
D-Ribitol-5P
1.1.1.56
2.7.1.47
1.1.1.405
2.7.7.40
CDP-ribitol
D-Ribulose
D-Ribulose-5P
2-Oxoglutarate
1.1.2.2
Pyruvate
Citrate cycle
Riboflavin metabolism
Pentose phosphate pathway
00040 5/19/17
(c) Kanehisa Laboratories

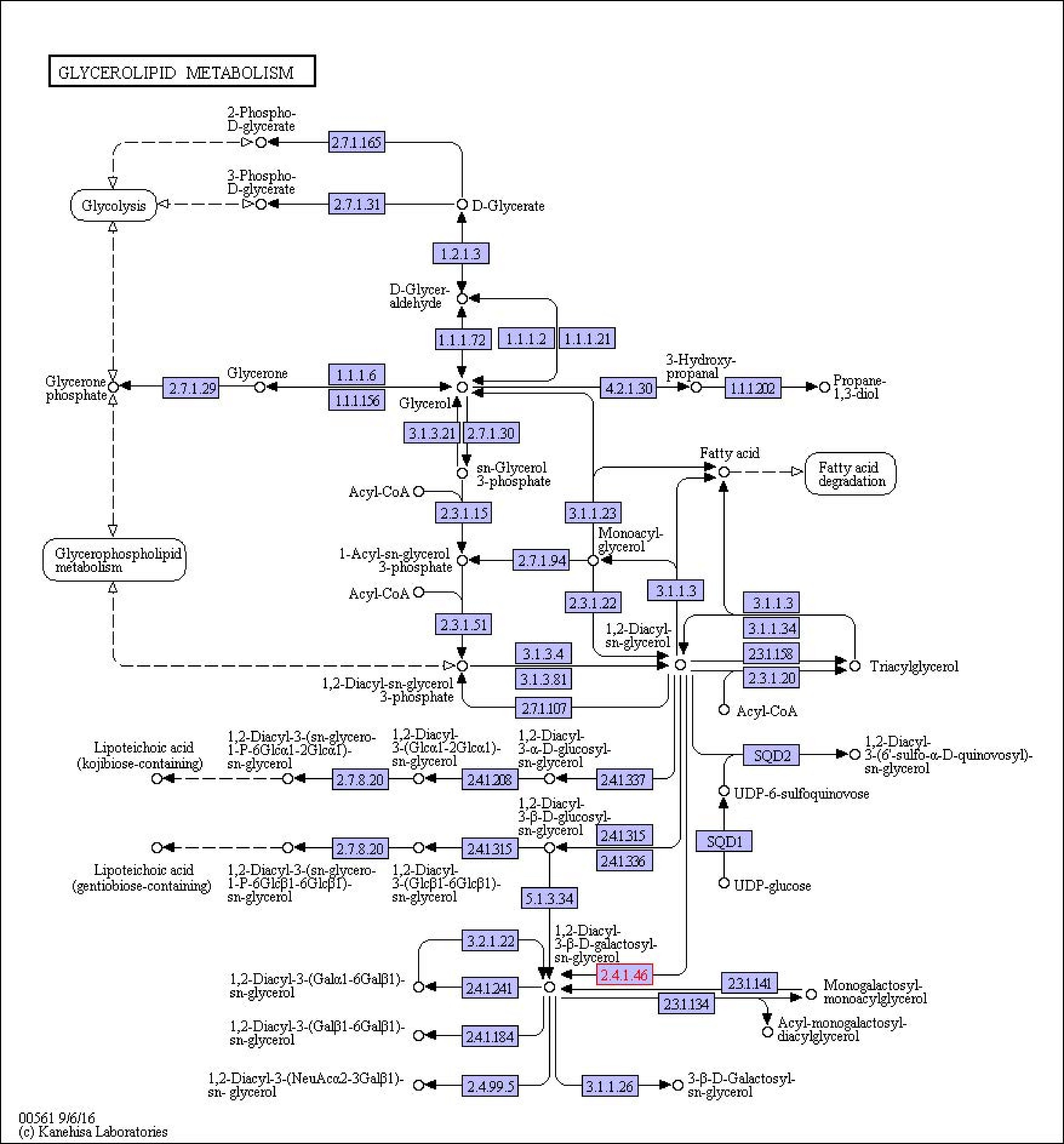

GLYCEROLIPID METABOLISM
2-Phospho-D-glycerate
2.7.1.165
3-Phospho-D-glycerate
Glycolysis
2.7.1.31
D-Glycerate
1.2.1.3
D-Glycer-aldehyde
1.1.1.72
1.1.1.2
1.1.1.21
Glycerone phosphate
2.7.1.29
Glycerone
1.1.1.6
1.1.1.156
Glycerol
4.2.1.30
3-Hydroxy-propanal
1.1.1.202
Propane-1,3-diol
3.1.3.21
2.7.1.30
sn-Glycerol 3-phosphate
Fatty acid
Fatty acid degradation
Acyl-CoA
2.3.1.15
3.1.1.23
Monoacyl-glycerol
Glycerophospholipid metabolism
1-Acyl-sn-glycerol 3-phosphate
2.7.1.94
3.1.1.3
2.3.1.22
Acyl-CoA
2.3.1.51
1,2-Diacyl-sn-glycerol
3.1.1.3
3.1.1.34
3.1.3.4
2.3.1.158
Triacylglycerol
3.1.3.81
2.3.1.20
1,2-Diacyl-sn-glycerol 3-phosphate
2.7.1.107
Acyl-CoA
Lipoteichoic acid (kojibiose-containing)
1,2-Diacyl-3-(sn-glycero-1-P-6Glcα1-2Glcα1)-sn-glycerol
2.7.8.20
1,2-Diacyl-3-(Glcα1-2Glcα1)-sn-glycerol
2.4.1.208
1,2-Diacyl-3-α-D-glucosyl-sn-glycerol
2.4.1.337
SQD2
1,2-Diacyl-3-(6'-sulfo-α-D-quinovosyl)-sn-glycerol
UDP-6-sulfoquinovose
1,2-Diacyl-3-β-D-glucosyl-sn-glycerol
2.4.1.315
SQD1
2.7.8.20
2.4.1.315
2.4.1.336
Lipoteichoic acid (gentiobiose-containing)
1,2-Diacyl-3-(sn-glycero-1-P-6Glcβ1-6Glcβ1)-sn-glycerol
1,2-Diacyl-3-(Glcβ1-6Glcβ1)-sn-glycerol
UDP-glucose
5.1.3.34
3.2.1.22
1,2-Diacyl-3-β-D-galactosyl-sn-glycerol
2.4.1.46
1,2-Diacyl-3-(Galα1-6Galβ1)-sn-glycerol
2.4.1.241
2.3.1.141
Monogalactosyl-monoacylglycerol
2.3.1.134
Acyl-monogalactosyl-diacylglycerol
1,2-Diacyl-3-(Galβ1-6Galβ1)-sn-glycerol
2.4.1.184
1,2-Diacyl-3-(NeuAcα2-3Galβ1)-sn- glycerol
2.4.99.5
3.1.1.26
3-β-D-Galactosyl-sn-glycerol
00561 9/6/16
(c) Kanehisa Laboratories

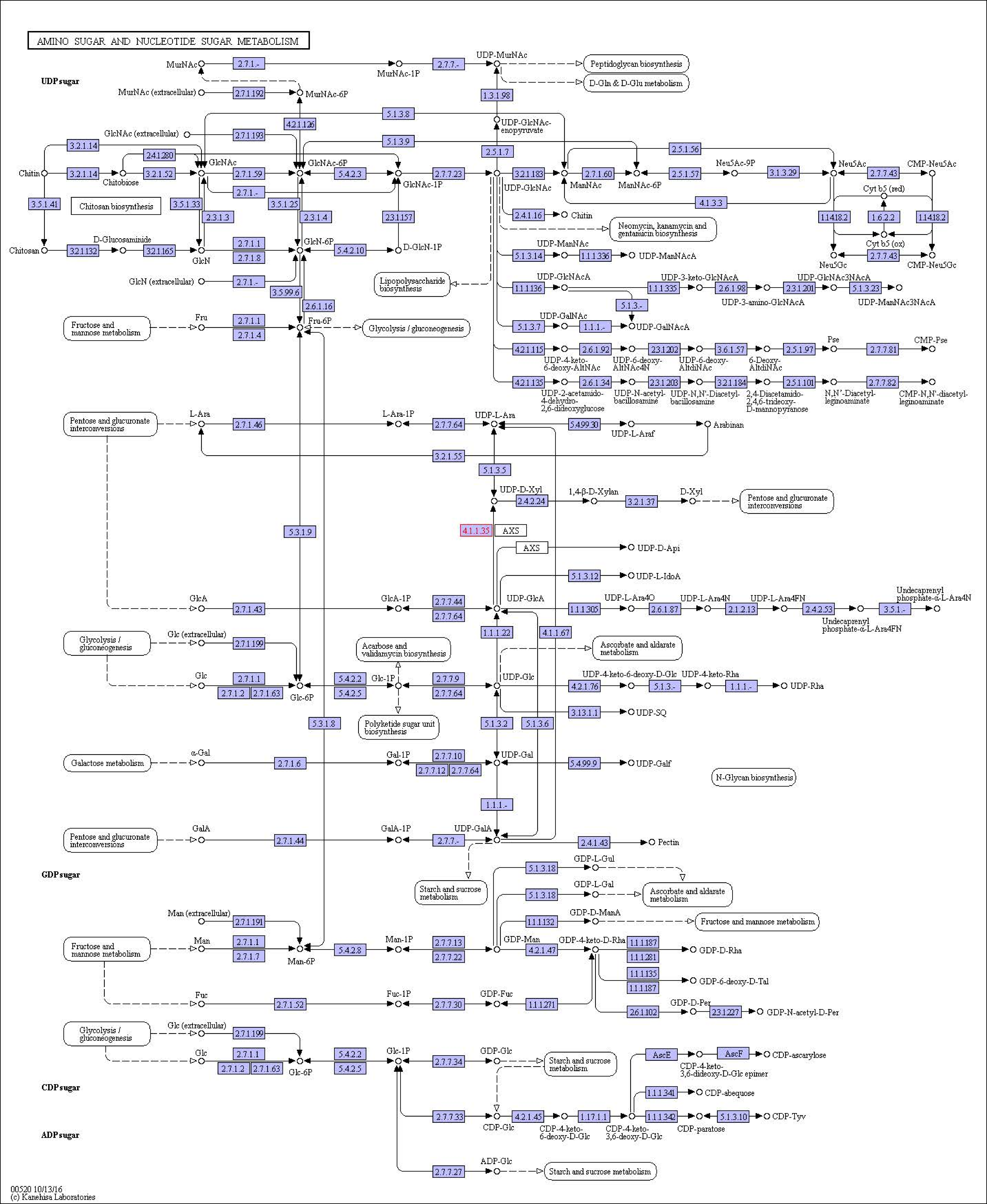
AMINO SUGAR AND NUCLEOTIDE SUGAR METABOLISM
UDP sugar
GDP sugar
CDP sugar
ADP sugar
MurNAc
2.7.1.-
MurNAc-1P
2.7.7.-
UDP-MurNAc
Peptidoglycan biosynthesis
D-Gln & D-Glu metabolism
MurNAc (extracellular)
2.7.1.192
MurNAc-6P
1.3.1.98
4.2.1.126
5.1.3.8
UDP-GlcNAc-enopyruvate
GlcNAc (extracellular)
2.7.1.193
5.1.3.9
2.5.1.7
3.2.1.14
2.4.1.280
2.5.1.56
Chitin
Chitobiose
3.2.1.52
GlcNAc
2.7.1.59
GlcNAc-6P
5.4.2.3
2.7.7.23
UDP-GlcNAc
3.2.1.183
ManNAc
2.7.1.60
ManNAc-6P
2.5.1.57
Neu5Ac-9P
3.1.3.29
Neu5Ac
2.7.7.43
CMP-Neu5Ac
2.7.1.-
GlcNAc-1P
4.1.3.3
Cyt b5 (red)
3.5.1.41
Chitosan biosynthesis
3.5.1.33
3.5.1.25
2.3.1.4
2.3.1.157
2.4.1.16
Chitin
1.14.18.2
1.6.2.2
2.3.1.3
Neomycin, kanamycin and gentamicin biosynthesis
Cyt b5 (ox)
UDP-ManNAc
D-Glucosaminide
2.7.1.1
GlcN-6P
Chitosan
3.2.1.132
3.2.1.165
GlcN
2.7.1.8
5.4.2.10
D-GlcN-1P
5.1.3.14
1.1.1.336
UDP-ManNAcA
Neu5Gc
CMP-Neu5Gc
UDP-GlcNAcA
UDP-3-keto-GlcNAcA
UDP-GlcNAc3NAcA
Lipopolysaccharide biosynthesis
1.1.1.136
1.1.1.335
2.6.1.98
2.3.1.201
5.1.3.23
GlcN (extracellular)
3.5.99.6
5.1.3.-
UDP-3-amino-GlcNAcA
UDP-ManNAc3NAcA
2.6.1.16
UDP-GalNAc
Fructose and mannose metabolism
Fru
2.7.1.4
Fru-6P
Glycolysis / gluconeogenesis
5.1.3.7
1.1.1.-
UDP-GalNAcA
Pse
CMP-Pse
4.2.1.115
2.6.1.92
2.3.1.202
3.6.1.57
2.5.1.97
2.7.7.81
UDP-4-keto-6-deoxy-AltNAc
UDP-6-deoxy-AltNAc4N
UDP-6-deoxy-AltdiNAc
6-Deoxy-AltdiNAc
4.2.1.135
2.6.1.34
2.3.1.203
3.2.1.184
2.5.1.101
2.7.7.82
UDP-2-acetamido-4-dehydro-2,6-dideoxyglucose
UDP-N-acetyl-bacillosamine
UDP-N,N'-Diacetyl-bacillosamine
2,4-Diacetamido-2,4,6-trideoxy-D-mannopyranose
N,N'-Diacetyl-legionaminate
CMP-N,N'-diacetyl-legionaminate
Pentose and glucuronate interconversions
L-Ara
2.7.1.46
L-Ara-1P
2.7.7.64
UDP-L-Ara
5.4.99.30
UDP-L-Araf
Arabinan
3.2.1.55
5.1.3.5
UDP-D-Xyl
2.4.2.24
1,4-β-D-Xylan
3.2.1.37
D-Xyl
4.1.1.35
AXS
UDP-D-Api
5.3.1.9
5.1.3.12
UDP-L-IdoA
Undecaprenyl phosphate-α-L-Ara4N
GlcA
2.7.1.43
GlcA-1P
2.7.7.44
UDP-GlcA
1.1.1.305
UDP-L-Ara4O
2.6.1.87
UDP-L-Ara4N
2.1.2.13
UDP-L-Ara4FN
2.4.2.53
3.5.1.-
Undecaprenyl phosphate-α-L-Ara4FN
1.1.1.22
4.1.1.67
Glc (extracellular)
2.7.1.199
Acarbose and validamycin biosynthesis
Ascorbate and aldarate metabolism
UDP-4-keto-6-deoxy-D-Glc
UDP-4-keto-Rha
Glc
2.7.1.2
2.7.1.63
Glc-6P
5.4.2.2
5.4.2.5
Glc-1P
2.7.7.9
UDP-Glc
4.2.1.76
UDP-Rha
3.13.1.1
UDP-SQ
5.3.1.8
Polyketide sugar unit biosynthesis
5.1.3.2
5.1.3.6
Galactose metabolism
α-Gal
2.7.1.6
Gal-1P
2.7.7.10
2.7.7.12
UDP-Gal
5.4.99.9
UDP-Galf
N-Glycan biosynthesis
GalA
2.7.1.44
GalA-1P
UDP-GalA
2.4.1.43
Pectin
Starch and sucrose metabolism
5.1.3.18
GDP-L-Gul
GDP-L-Gal
Man (extracellular)
2.7.1.191
1.1.1.132
GDP-D-ManA
Man
2.7.1.7
Man-6P
5.4.2.8
Man-1P
2.7.7.13
2.7.7.22
GDP-Man
4.2.1.47
GDP-4-keto-D-Rha
1.1.1.187
1.1.1.281
GDP-D-Rha
1.1.1.135
GDP-6-deoxy-D-Tal
Fuc
2.7.1.52
Fuc-1P
2.7.7.30
GDP-Fuc
1.1.1.271
GDP-D-Per
2.6.1.102
2.3.1.227
GDP-N-acetyl-D-Per
GDP-Glc
2.7.7.34
AscE
AscF
CDP-ascarylose
CDP-4-keto-3,6-dideoxy-D-Glc epimer
1.1.1.341
CDP-abequose
2.7.7.33
CDP-Glc
4.2.1.45
1.17.1.1
1.1.1.342
5.1.3.10
CDP-Tyv
CDP-4-keto-6-deoxy-D-Glc
CDP-4-keto-3,6-deoxy-D-Glc
CDP-paratose
ADP-Glc
2.7.7.27
00520 10/13/16
(c) Kanehisa Laboratories

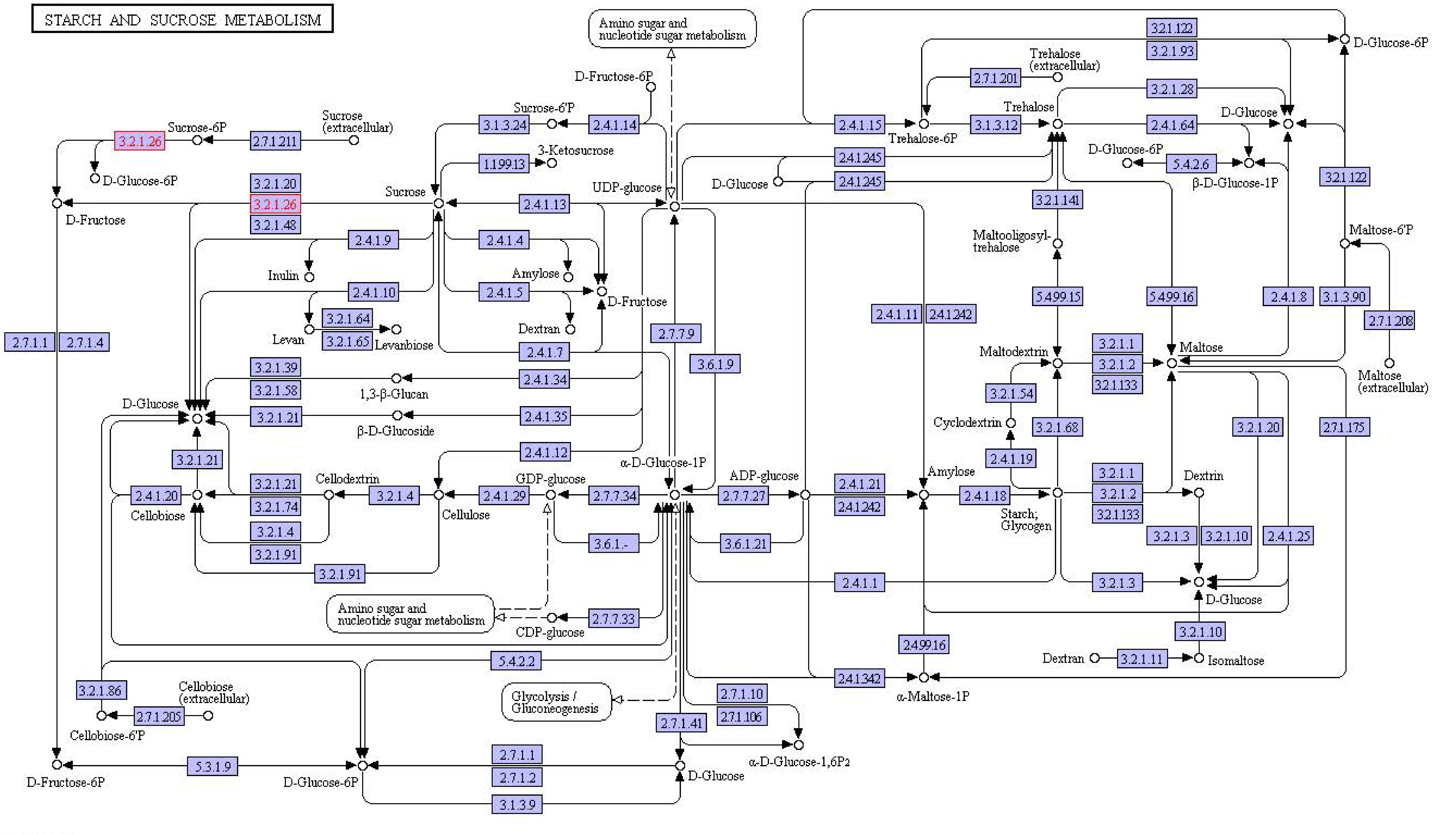

00500 2/9/17
(c) Kanehisa Laboratories
